# Cancer Cell‐Specific Fluorescent Prodrug Delivery Platforms

**DOI:** 10.1002/advs.202207768

**Published:** 2023-04-07

**Authors:** Siyue Ma, Ji Hyeon Kim, Wei Chen, Lu Li, Jieun Lee, Junlian Xue, Yuxia Liu, Guang Chen, Bo Tang, Wei Tao, Jong Seung Kim

**Affiliations:** ^1^ The Youth Innovation Team of Shaanxi Universities Shaanxi Key Laboratory of Chemical Additives for Industry College of Chemistry and Chemical Engineering Shaanxi University of Science & Technology Xi'an 710021 China; ^2^ Key Laboratory of Emergency and Trauma, Ministry of Education College of Emergency and Trauma Hainan Medical University Haikou 571199 China; ^3^ Department of Chemistry Korea University Seoul 02841 South Korea; ^4^ Center for Nanomedicine and Department of Anesthesiology Brigham and Women's Hospital Harvard Medical School Boston MA 02115 USA; ^5^ College of Chemistry Chemical Engineering and Materials Science Key Laboratory of Molecular and Nano Probes Ministry of Education Collaborative Innovation Center of Functionalized Probes for Chemical Imaging in Universities of Shandong Institutes of Biomedical Sciences Shandong Normal University Jinan 250014 China

**Keywords:** cancer specific targeting, drug delivery system, fluorescent prodrug, nanoparticles, tumor therapy

## Abstract

Targeting cancer cells with high specificity is one of the most essential yet challenging goals of tumor therapy. Because different surface receptors, transporters, and integrins are overexpressed specifically on tumor cells, using these tumor cell‐specific properties to improve drug targeting efficacy holds particular promise. Targeted fluorescent prodrugs not only improve intracellular accumulation and bioavailability but also report their own localization and activation through real‐time changes in fluorescence. In this review, efforts are highlighted to develop innovative targeted fluorescent prodrugs that efficiently accumulate in tumor cells in different organs, including lung cancer, liver cancer, cervical cancer, breast cancer, glioma, and colorectal cancer. The latest progress and advances in chemical design and synthetic considerations in fluorescence prodrug conjugates and how their therapeutic efficacy and fluorescence can be activated by tumor‐specific stimuli are reviewed. Additionally, novel perspectives are provided on strategies behind engineered nanoparticle platforms self‐assembled from targeted fluorescence prodrugs, and how fluorescence readouts can be used to monitor the position and action of the nanoparticle‐mediated delivery of therapeutic agents in preclinical models. Finally, future opportunities for fluorescent prodrug‐based strategies and solutions to the challenges of accelerating clinical translation for the treatment of organ‐specific tumors are proposed.

## Graphical abstract

A general overview of guides to present the targeted fluorescent prodrugs platforms that efficiently accumulate in tumor cells of diverse organs (lung cancer, liver cancer, cervical cancer, breast cancer, glioma, and colorectal cancer), which provides practical perspectives on the strategies behind the engineered nanoparticle platforms that are assembled from targeted fluorescence prodrugs.

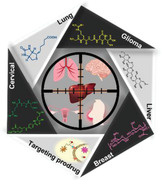

## 1 Introduction

Targeting cancer cells with high specificity plays a pivotal role in precision therapy.^[^ ^1^ ^]^ Although various anticancer drugs have been developed, the clinical situation remains serious: drugs kill not only cancer cells but also many normal cells as well. The patient's immunity declines, making the body vulnerable and unable to fight back when cancer cells resurge.^[^ ^2^ ^]^ Furthermore, cancer cells are heterogeneous, making drug targeting and treatment particularly challenging.^[^ ^3^ ^]^

**Figure 1:**
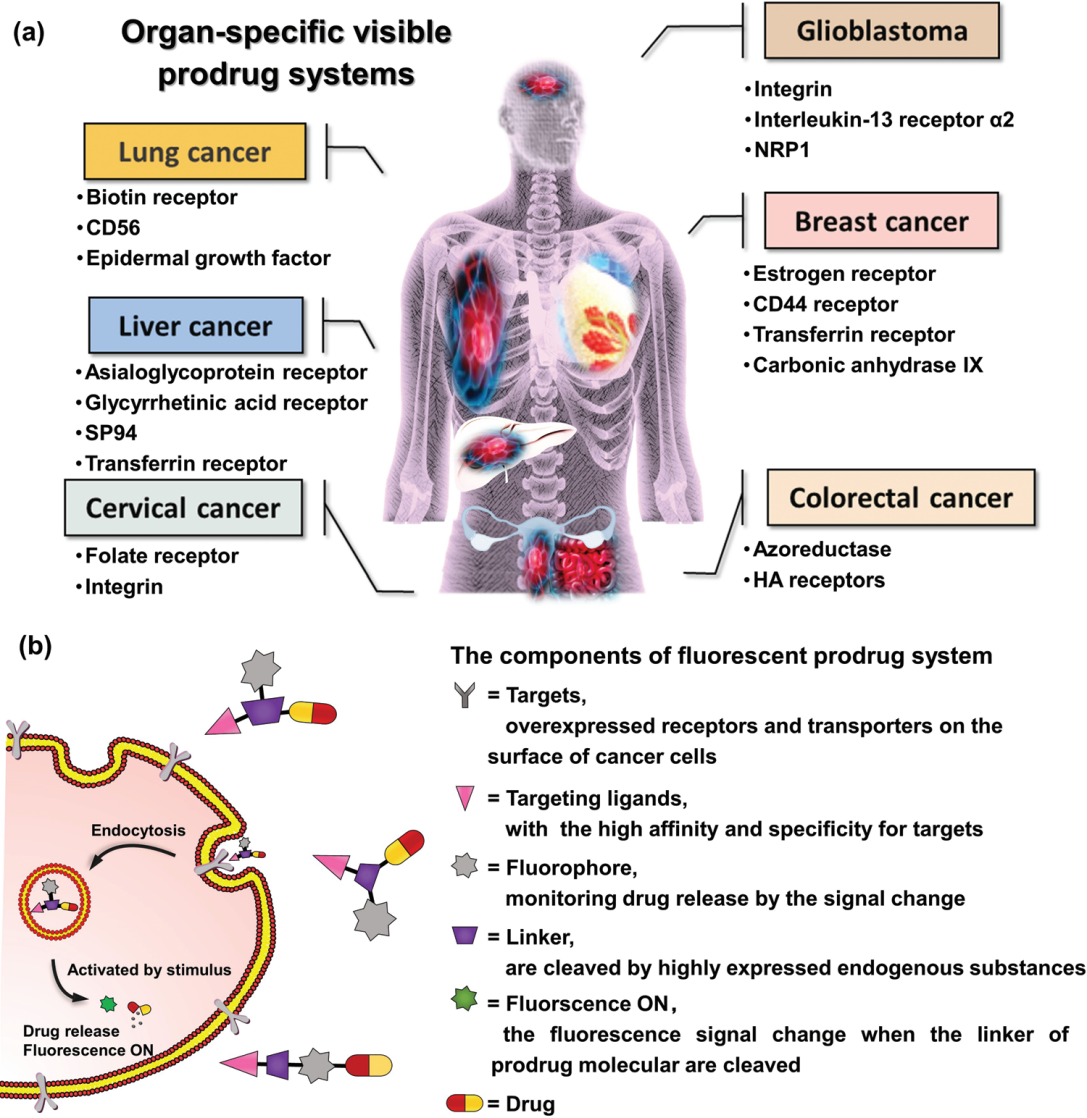
Overview of targeted fluorescent prodrug system. a) Organ‐specific tumor cell surface receptors and integrins can be used to design innovative fluorescent prodrug systems, including lung cancer, liver cancer, cervical cancer, breast cancer, glioblastoma cancer, and colorectal cancer. b) The components of the targeted fluorescent prodrug systems and how the fluorescent prodrug system can be used to target cancer cells for stimuli‐responsive drug release.

Improving drug targeting efficiency by harnessing cancer cell‐specific properties holds great promise for tumor therapies. Of particular significance, unlike normal cells, cancer cells divide rapidly and indiscriminately.^[^ ^4^ ^]^ In essence, it is the rapid flow of nutrients through the overexpressed receptors and transporters on the surface of cancer cells that causes such wanton cell division. Therefore, the following important criteria for the use of cell receptors and transporters for drug targets should be adequately considered. First, the expression level of cell receptors and transporters on cancer cells should be higher than that of normal cells. Second, the expression level on cancer cells must be sufficient for the drugs to be adequately delivered through the surface receptors or transporters. Third, the overexpressed receptors and transporters should exist on the surface of tumor cell membrane, not in the cytoplasm or nucleus,^[^ ^5^ ^]^ which allows the administered drugs to effectively contact these receptors. The following cell receptors and transporters that fulfill these considerations are suitable targets for drug design: lung cancer: biotin receptors, epidermal growth factor receptor (EGFR), CD56 receptors; liver cancer: asialoglycoprotein receptor (ASGPR), glycyrrhetinic acid receptor (GAR), SP94, transferrin receptor (TfR); cervical cancer: folate receptors (FRs) and integrins; breast cancer: estrogen receptors (ER), carbonic anhydrase (CAIX), etc.; glioma: integrin, interleukin‐13 receptor *α*2 (IL‐13R*α*2), neuropilins (NRPs), glioma‐specific chloride ion channel (GCC); and colorectal cancer: azoreductase, hyaluronic acid (HA) receptors (**Figure** **1**a). Extensive efforts have been made to design and develop innovative targeted drugs or nanocarriers, such as small molecules,^[^ ^6^ ^]^ self‐assembling systems,^[^ ^7^ ^]^ polymeric nanoparticles,^[^ ^8^ ^]^ metal–organic frameworks hybrid porous materials,^[^ ^9^ ^]^ and supramolecular host–guest systems, that can target these tumor cell‐specific surface receptors to improve the intracellular accumulation.

In addition to efficient accumulation in tumor cells, monitoring drug release in in vivo models and living cells with spatiotemporal accuracy remains challenging. The use of targeted fluorescent prodrugs in conjunction with noninvasive fluorescence imaging technology could address this challenge.^[^ ^10^ ^]^ In principle, the ideal fluorescent targeted prodrug systems consist of the following four components: targeting ligands, cleavable linkers, imageable fluorophores, and antineoplastic drugs (Figure 1b).^[^ ^11^ ^]^ For targeting ligands, the selectivity for overexpressed cell membrane receptors will help the prodrug specifically target cancer cells and avoid toxicity to healthy tissues, of course, the optimal affinity, and functional‐group availability are also important for ligands.^[^ ^12^ ^]^ In addition, the targeting ligand should contain a derivable functional moiety (e.g., carboxyl, amino, hydroxyl, thiol) that could be conjugated with the fluorescent dyes and anticancer drugs through a cleavable linker to realize the visible imaging and drug release. Importantly, the linkers must remain intact until the fluorescent prodrug system reaches the tumor cell, but it should be cleavable when captured and internalized by cancer cells.^[^ ^13^ ^]^ Therefore, the moieties (such as amides, esters, or disulphides) that are cleaved by highly expressed endogenous substances, for example, reactive oxygen species (ROS), reactive sulfur species (RSS), acidic pH, enzymes, etc., or external stimuli, such as light, can be used as linkers.^[^ ^14^ ^]^ Generally, an elimination cascade needs to be designed in the linker in order to ensure that the atoms in the spacer do not attach to the released drug. Additionally, the fluorophores are used to monitor the amount and location of the drug released in real‐time by the change in fluorescence intensity and signal, so as to evaluate the effect of drug delivery. The traditional fluorophores (coumarin, ethidium, BODIPY, fluorescein, etc.) with short wavelength fluorescence emission are subject to interference by the autofluorescence of organisms, which is still limited in their clinical application. Therefore, the use of fluorophores with near‐infrared (NIR) emission or two‐photon property (cyanine, xanthene‐based NIR chromophores, etc.) has shown great promise in bioimaging. Importantly, the size of fluorescent prodrugs that may affect the pharmacokinetic profiles and drug accumulation efficiency should also be considered. In general, following intravenous administration the circulating targeted fluorescent prodrugs (e.g., polymers and liposomes) could first accumulate in tumor tissues through the enhanced permeability and retention (EPR) effects. Subsequently, the targeted fluorescent prodrugs could be uptake by tumor cells through a receptor‐mediated endocytosis manner. Finally, the intracellular targeted fluorescent prodrugs might release the “active” drugs for cell killing activated by the endogenous/external stimuli mentioned before. To maximize cancer cell killed, careful selection of an appropriate drug is essential for the design of fluorescent prodrug delivery platforms. It is tempting to think that this potency limitation can be addressed by attaching more therapeutic warheads to the same targeting ligand, but developing a single drug molecule that is ten times more potent is often more effective than attaching ten of the same drug molecules. For drug delivery following receptor‐mediated endocytosis, the released drug must leave the closed endosome and be therapeutically activated. Most established chemotherapeutic agents are already sufficiently membrane permeable to demonstrate efficacy when added to intact cells. Therefore, the selected drugs for fluorescent prodrug delivery platforms are all known to have strong efficacy, such as camptothecin (CPT), gemcitabine (GMC), doxorubicin (DOX), etc.

In this Review, we discuss and present novel perspectives on 1) the rational design of fluorescent prodrug systems that can selectively target tumor cells in different organs through surface receptors to improve therapeutic efficacy while minimizing off‐target side effects; 2) the design of nanoparticle‐based platforms that can improve the pharmacokinetics of encapsulated fluorescent prodrugs and enhance their tumor accumulation and bioavailability through receptor‐mediated endocytosis and release mechanisms that respond to tumor microenvironments, including ROS, RSS, acidic pH, or enzymes. Most importantly, the targeted fluorescent prodrugs that integrate fluorophores, linkers, targeting ligands, and drugs may offer the potential of molecule targeting, fluorescence triggering and stimuli‐responsive drug release in an individual theranostic system, and thereby report both the fate and the therapeutic efficacy of the released drugs.

## 2 Fluorescent Molecular Prodrugs for Lung Cancer Treatment

Biotin and CD56 receptors are overexpressed in lung cancer cells, and therefore could serve as ideal targets in the design of innovative prodrug systems.^[^ ^15^ ^]^ In addition, EGFR, a transmembrane receptor tyrosine kinase protein, is associated with cancer initiation, progression, and poor prognosis of lung cancers.^[^ ^16^ ^]^ Stimuli‐responsive fluorophores that can be triggered to fluoresce “ON” have been bonded to these receptors, yielding fluorescent prodrugs for lung cancer treatment (**Table** **1**).

**Table 1:** Targeted fluorescent prodrugs for lung cancer

Targets	Targeting ligand	Targeted cell	Fluorophore	Activator	Refs.
Biotin receptor	Biotin	A549	Coumarin	GSH	[18]
			BODIPY	Thiols	[19]
			Ethidium	H2O2	[20]
			Quinone propionic acid	NQO1	[21]
			Tetraphenylene	pH 5.5	[22]
			BODIPY	GSH	[23]
CD56 receptor	Capreomycin	H446 cells	ICG	GSH	[24]
EGFR	Gefitinib	A549	azo‐BODIPY	GSH	[26]
	Gefitinib	A549	Xanthene‐based NIR chromophore	GSH	[27]
	Erlotinib	A549	Quercetin	GSH	[28]

### 2.1 Biotin Receptor Targeting

**Figure 2:**
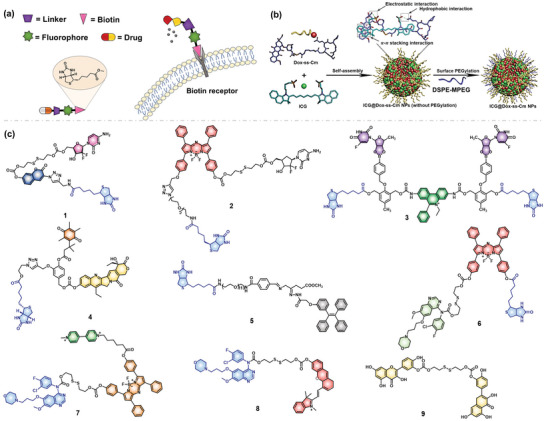
Fluorescent prodrug systems for lung cancer. a) Strategies of biotin receptor as a target for lung cancer. b) The rationale of CD56 receptor as a target for lung cancer and the strategies of CD56 receptor as a target. Reproduced with permission.^[^ ^24^ ^]^ Copyright 2020, American Chemical Society. c) Chemical structures of fluorescent prodrugs for lung cancer.

Biotin receptors are overexpressed in nonsmall cell lung cancer (NSCLC). Through receptor‐mediated endocytosis, prodrug systems containing receptor ligands accelerate drug accumulation in cancer cells and reduce both toxicity and side effects (**Figure** **2**a).^[^ ^17^ ^]^

Kim's group put great effort into developing prodrugs (Figure 2c, compounds 1–6) targeting biotin receptor‐positive cells (A549), also providing a useful strategy for specific drug delivery in tumors with imaging monitoring. The cleavage of the disulfide bond (S—S) is an efficient strategy for the construction of “OFF‐ON” fluorescent probes or prodrugs. For example, the fluorescence of a fluorophore conjugated to a drug through an S—S bond is quenched via a photoinduced electron transfer mechanism. Reacting with glutathione (GSH), the S—S bond is cleaved and undergoes an intramolecular nucleophilic attack at the carbamate moiety to yield a five‐membered ring, both releasing the drug and restoring fluorescence. For example, a compound connects coumarin (fluorescent reporter), GMC(drug), and a biotin unit (cancer targeting) by a cleavable disulfide bond.^[^ ^18^ ^]^ Researchers found that the biotin moiety can serve as a targeting moiety for lung cancer cells. Moreover, because NIR fluorophores are capable of deep tissue penetration, the same group reported an activatable NIR (720 nm) fluorescent prodrug containing the biotin unit that could be cleaved to release GMC.^[^ ^19^ ^]^ In addition, they presented a highly efficient mitochondria‐targeting antitumor prodrug containing 5’‐deoxy‐5‐fluorouridine (drug) and biotin, which was activated by endogenous mitochondrial‐overexpressed H_2_O_2_ to release the drug.^[^ ^20^ ^]^ That in vivo xenograft tumor model was used to demonstrate that treatment with this prodrug inhibited tumor progression significantly. To achieve the dual goal of accurate diagnosis and treatment of cancer, a prodrug containing an anti‐cancer drug (7‐ethyl‐10‐hydroxycamptothecin, SN‐38) for inhibiting topoisomerase I in cell nuclei was synthesized.^[^ ^21^ ^]^ In this prodrug, hydroquinone could be triggered by NQO1, and biotin could serve as cancer cell‐targeting moiety. The fluorescence signal was used to monitor drug release and observe the apoptosis of cancer cells that have high expression of biotin receptors and high levels of NQO1. This design demonstrated that the enzyme‐triggered prodrug could inhibit cancer growth. Moreover, an intracellular dual fluorescent bio‐probe containing tetraphenylene (TPE) and methyl aminolevulinate was reported;^[^ ^22^ ^]^ the compound selectively illuminated cancer cells with blue fluorescent TPE and red fluorescent photosensitizer protoporphyrin IX (PpIX). In addition, owing to the endogenous generation and accumulation of PpIX inside lung cancer cells, photodynamic ablation can be effectively performed with light irradiation to enhance the therapeutic effect. The design of such endogenous dual fluorescence prodrugs may provide a feasible approach for PDT. In addition, an innovative cancer‐targeting prodrug with the integration of real‐time fluorescence visualization was reported, in which biotin was used to guide gefitinib to cancer cells.^[^ ^23^ ^]^ This prodrug accumulated in cancer lesions with a better and more efficacious therapeutic outcome compared with gefitinib alone. In addition, the drug release in vivo was successfully monitored using this prodrug platform, which displayed great potential for other clinical applications.

### 2.2 CD56 Receptor Targeting

CD56 is overexpressed on the surface of small cell lung cancer (SCLC) cells, and the positive rate of pathology detection is significantly higher than that of other neuroendocrine biomarkers. Capreomycin has a high affinity for CD56 receptors and therefore can serve as a specific targeting ligand for CD56 (Figure 2b).^[^ ^24^ ^]^ The self‐distinguishing and multiresponsive nanoagents (ICG@Dox‐ss‐Cm) nanoparticles whose surface was modified with capreomycin for the specific delivery of therapeutics to SCLC cells. The drug delivery system also released its therapeutic payloads in response to multiple stimuli, including intracellular lysosomal acidity, GSH, and an external NIR laser. In addition, the precise guidance, assisted by NIRF/PA dual‐modal imaging, made possible the highly efficient elimination of SCLC in only one cycle of therapy, owing to the high optical sensitivity of fluorescence imaging and the excellent tissue penetration of photoacoustic imaging.

### 2.3 Epidermal Growth Factor Receptor Targeting

EGFR is an attractive target for cancer therapy, and EGFR‐targeted inhibitors such as gefitinib and erlotinib have been developed as targeting agents for NSCLC.^[^ ^25^ ^]^ A new prodrug **7** containing a NIR azo‐BODIPY fluorophore, a tumor‐targeting ligand polyamine analog, as well as an EGFR tyrosine kinase inhibitor gefitinib was reported for NSCLC therapy (Figure 2c).^[^ ^26^ ^]^ The polyamine analog selectively delivered the prodrug to tumor cells, enhanced the efficacy of gefitinib and reduced adverse effects. Moreover, this fluorescent prodrug could be used to monitor the real‐time release of the drug in vivo. Gefitinib has also been used in a nanodrug platform containing a serine‐threonine protein kinase (Akt) inhibitor (Celastrol).^[^ ^27^ ^]^ In addition, another nanodrug **8** conjugated with a NIR fluorophore by a cleavable disulfide bond was reported (Figure 2c). Such a nanodrug platform was capable of (i) efficiently accumulating therapeutic drugs in the tumor region of NSCLC‐bearing mice, (ii) releasing the drugs for tumor inhibition, and (iii) optoacoustic imaging via the fluorescent dye. In addition, the same group reported a nanoprodrug system **9** that was prepared by a disulfide‐facilitated assembly strategy (Figure 2c).^[^ ^28^ ^]^ Among the nanoprodrug systems, erlotinib actively blocked EGFR tyrosine kinase, and quercetin not only contributed to the inhibition of downstream EGFR signaling but also demonstrated strong aggregation‐induced fluorescence to image drug release. The study indicated that treatment with nanoprodrug system **9** induced a high percentage (28.5–75.6%) of apoptosis, which was higher than that of the erlotinib (13.1–58%), quercetin (4.1–22.8%), and QSSQ (8.6–35.7%). This rationally designed nanoprodrug system achieved high‐efficacy combination therapy for NSCLC tumors.

## 3 Fluorescent Prodrug Systems for Liver Cancer

Liver cancers can occur as primary cancer or as secondary metastatic cancer by spreading from other body parts.^[^ ^29^ ^]^ Hepatocellular carcinoma (HCC) accounts for more than 90% of all nonmetastatic primary liver cancers.^[^ ^30^ ^]^ Overexpression of certain receptors for liver cancer enables selective imaging and drug delivery. ASGPR and GAR are promising targets for drug delivery in liver cancer (**Table** **2**).

**Table 2:** Targeted fluorescent prodrugs for liver cancer

Targets	Targeting ligand	Targeted cell	Fluorophore	Activator	Refs.
ASGPR	Gal	HepG2 cells	CPT	GSH	[36]
	lactose		CPT	GSH	[37]
	Pullulan	MHCC‐97H cells	IR780	NIR laser	[38a]
GAR	GA	HepG2 cells	DOX	Carboxylesterase	[43a]
			FITC		[44]
	SP94	HCC‐LM3, BEL‐7402	DiR	Acidic pH	[47]
TfR	Tf	SMMC‐7721 cells	Cy5.5		[51]
			DOX	GSH	[52]

### 3.1 Asialoglycoprotein Receptor Targeting

**Figure 3:**
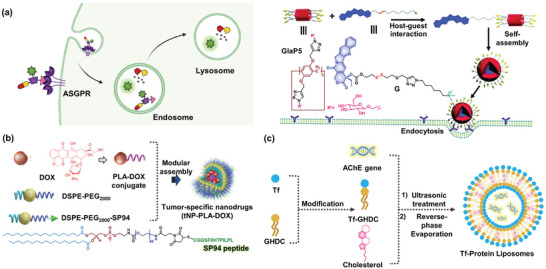
Fluorescent prodrug systems for liver cancer. a) *β*‐D‐Gal modified liver cancer targeting supramolecular nanoparticle prodrug. Reproduced with permission.^[^ ^36^ ^]^ Copyright 2017, Royal Society of Chemistry. b) Chemical structure and synthetic scheme of SP94 fabricated DSPE‐PEG‐SP94 NPs. Reproduced with permission.^[^ ^47^ ^]^ Copyright 2018, American Chemical Society. c) Preparation of Tf liposome and illustration of prodrug activation in the liver cancer cell.

ASGPR, a liver‐specific lysosome‐targeting receptor, is the most common target for drug delivery systems owing to its high expression on HCC but low expression outside hepatocytes.^[^ ^31^ ^]^ ASGPR has high specificity and affinity for galactose (Gal), galactosamine, and pullulan.^[^ ^32^ ^]^ Therefore, these structures were selected as targeting ligands for ASGPR (**Figure** **3**a).

Since the entry of substances through ASGPR occurs via clathrin‐mediated endocytosis, ASGPR exhibits a high affinity for carbohydrates, particularly Gal, *N*‐acetylgalactosamine (GalNAc), and glucose.^[^ ^33^ ^]^ Gal is a representative target of ASGPR in HCC which has been extensively exploited for targeted imaging.^[^ ^34^ ^]^ For example, a fluorescent probe (DCDHF‐*β*gal) for in vivo imaging of HCC was developed leveraging the NIR fluorophore dicyanomethylenedihydrofuran (DCDHF).^[^ ^35^ ^]^ The tumor‐specific targeting ability of DCDHF‐*β*gal was investigated using a HepG2 tumor‐bearing xenograft mouse model. The utilization of DCDHF‐*β*gal for HCC imaging showed great promise because HepG2 cells, overexpressing ASGPR, can facilitate the uptake and further cleavage of DCDHF‐*β*gal by *β*‐galactosidase, enabling DCDHF‐*β*gal to release a free and redshifted fluorophore. Furthermore, Gal‐based ASGPR‐targeting fluorescence prodrug‐delivery platform was developed as a supramolecular nanoparticle‐based theragnostic system,^[^ ^36^ ^]^ i.e., an amphiphilic triblock copolymer containing Gal as an ASGPR‐targeting ligand, (2‐diisopropylamino) ethyl (DPA) as the hydrophobic core and acid sensor, and diazeniumdiolate as a NO donor. Importantly, this prodrug system can release cytotoxic nitric oxide (NO) in a GSH‐responsive manner to kill HCC cells. Lactose, which is a disaccharide composed of glucose and Gal, can be efficiently endocytosed through the ASGPR in HCC.^[^ ^37^ ^]^ For example, a self‐assembled CPT prodrug nanoparticle (CPT‐S‐S‐LA) with lactose targeting group can release the CPT drug in GSH‐overexpressing cancer cells. Drug release was evaluated by distinct fluorescence of CPT; the fluorescence intensity was much higher in HepG2 cells treated by CPT‐S‐S‐LA nanoparticles than in the normal HUVEC cells in vitro, attributed to the LA targeting unit. Moreover, the experiment of cytotoxicity and apoptosis showed that the percentage of apoptotic HepG2 cells in the CPT‐S‐S‐LA nanoparticles treatment group was 17.96% (higher than that of 14.10% with the free CPT), which demonstrated that the CPA‐S‐S‐LA nanoparticles improved the antitumor efficacy.

Pullulan is a water‐soluble, nontoxic, nonimmunogenic, nonmutagenic, and noncarcinogenic polysaccharide that has a specific affinity for the overexpressed ASGPR on HCC.^[^ ^38^ ^]^ A pullulan‐modified nanoparticle to target HCC through the combination of photothermal/photodynamic and chemotherapy was developed by using IR780 and paclitaxel dual cargo‐loaded nanoparticles.^[^ ^39^ ^]^ The targeting ability of pullulan was evaluated using a fluorescence photosensitizer (IR780) in PDFI, demonstrating that the cellular uptake of pullulan‐modified nanoparticles was better than that of pullulan‐free nanoparticles. Recently, beyond multifunctional fluorescent prodrugs, ASGPR‐targeted therapeutic modalities have been actively studied on LYsosome TArgeting Chimeras (LYTACs),^[^ ^40^ ^]^ which decomposes target proteins in lysosomes after ASGPR‐mediated endocytosis.

### 3.2 Glycyrrhetinic Acid Receptor (GAR) Targeting

The abundance of GAR on the surface of liver cancer cells is 1.5‐ to 4‐times higher than that on normal tissues,^[^ ^41^ ^]^ which has provided a strong rationale to develop glycyrrhetinic acid‐modified nanoparticles for HCC treatment. For example, glycyrrhetinic acid‐modified chitosan/poly(ethylene glycol) nanoparticles have been developed to treat HCC tumor‐bearing mice.^[^ ^42^ ^]^ In addition, other glycyrrhetinic acid‐modified theranostic materials for HCC treatment have been developed.^[^ ^43^ ^]^ For example, adenine‐loaded nanoparticles were reported whose surface was modified with GA and HA (Ade/GA‐HA) for liver targeting.^[^ ^44^ ^]^ As GA is a pentacyclic triterpenoid glycoside exhibiting hydrophobic characteristics, the water solubility concern was resolved by using hydrophilic HA, which is a CD44 receptor‐binding acid mucopolysaccharide. For in vitro imaging of HepG2 cells, HA was labeled with fluorescein isothiocyanate (FITC) to yield FITC‐labeled GA‐HA for incorporation into originally produced GA‐HA NPs. For in vivo imaging of HepG2 xenograft BALB/c nude mouse model, a NIR fluorophore (DiR) was loaded into Ade/GA‐HA NP, thereby demonstrating liver targeting and cellular uptake enhancement.

### 3.3 SP94 Targeting Peptide

Because SP94 peptide (sequence: SFSIIHTPILPL) shows a high specificity for HCC but not for other normal cells or cancer cells, this cost‐effective 12‐residue peptide has been widely utilized in drug delivery systems to enhance HCC targeting.^[^ ^45^ ^]^ For example, HCC cell‐targeting protein cage was developed by engineering a heat shock protein 90 (Hsp 90) cage surface using SP94 peptides.^[^ ^46^ ^]^ It was manifested that binding the protein cage at the N‐terminus of peptides was advantageous than the C‐terminus for efficient cell binding. In addition, an SP94‐modified diblock copolymer nanoparticle was developed by assembling with PLA‐DOX prodrug (Figure 3b).^[^ ^47^ ^]^ The drug release profile was quantified according to the fluorescence of the released DOX. Similarly, an SP94 peptide‐modified nanodrug (SP94‐PS‐DOX) containing DOX was developed and tested in orthotopic HCC xenograft models.^[^ ^48^ ^]^ These reports support the promise of SP94 peptide as a targeting ligand for improved antitumor efficacy.

### 3.4 Transferrin Receptor Targeting

Transferrin (Tf) is an endogenous protein that transports iron into cells through the cell membrane's TfR.^[^ ^49^ ^]^ There is a growing body of evidence that TfR expression in HCC is much higher than that in healthy tissues because liver cancer cells show upregulated iron metabolism.^[^ ^50^ ^]^ Therefore, Tf has been utilized as a specific tumor‐guiding unit to improve the accumulation of targeted fluorescent prodrugs in tumor tissues. Since Tf is a hydrophilic glycoprotein, when amphiphilic Tf‐glycidyl hexadecyl dimethylammonium chloride (GHDC) is prepared, a liposome formed with Tf on the outer surface can interact and facilitate uptake by TfR (Figure 3c).^[^ ^51^ ^]^ For example, a Dox‐loaded polycarbonate‐based nanoparticle (Tf‐Ps‐Dox) that could be guided by Tf was developed.^[^ ^52^ ^]^ Tf‐Ps‐Dox not only effectively accumulated in the SMMC‐7721 tumor‐bearing mice with the help of Tf targeting ligand but also achieved desirable therapeutic efficacy in an orthotopic SMMC‐7721 tumor‐bearing mouse model.

## 4 Fluorescent Prodrug Systems for Cervical Cancer

Cervical cancer accounts for 10–15% of cancer‐related deaths among women worldwide.^[^ ^53^ ^]^ Typically, FRs are overexpressed on the surface of cervical cancer cells (e.g., HeLa) but are deficient in other types of cancer cells.^[^ ^54^ ^]^ Therefore, FRs have served as the unique receptors for cervical cancer cell targeting. In addition, unique cell surface integrins are often used as therapeutic targets for the development of cervical cancer prodrug systems.^[^ ^55^ ^]^ Of note, in addition to the commonly used fluorophores, some fluorescent chemotherapeutic drugs, such as CPT and DOX, have been used to visually monitor the efficiency of drug release for cervical cancer treatment (**Table** **3**).

**Table 3:** Targeted fluorescent prodrugs for cervical cancer

Targets	Targeted units	Targeted cells	Fluorophores	Activator	Refs.
FR	FA	HeLa cell	CPT	GSH	[58]
			DOX	Acidic pH	[59]
			DOX	Acidic pH	[60]
Integrin *α*_v_ *β*_3_	RGD peptide	HeLa cell	Ru‐Complex	Acidic pH	[56]

### 4.1 Folate Receptor Targeting

**Figure 4:**
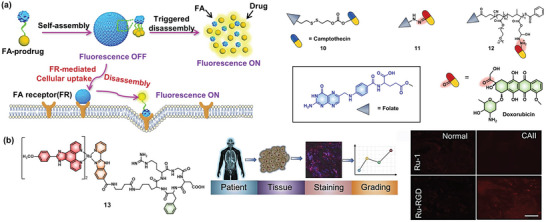
Fluorescent prodrug systems for cervical cancer. a) Strategy of folate receptor as target for cervical cancer. b) Structure of the prodrug **13** and its rapid tumor diagnosis in clinical specimens. Reproduced with permission.^[^ ^56^ ^]^ Copyright 2019, Elsevier.

A 2015 study demonstrated that the modification of folate can effectively improve the uptake of the prodrug system in FR‐overexpressing HeLa cells through FR‐mediated endocytosis (**Figure** **4**a).^[^ ^57^ ^]^ In addition, a GSH‐responsive small‐molecule prodrug **10** delivery system containing a folate‐targeting ligand and the hydrophobic antitumor drug CPT was reported.^[^ ^58^ ^]^ This prodrug has a high CPT loading efficacy (36.8 wt%) and exhibits greater specificity and cytotoxicity for KB tumor cells (FR positive) compared to that of A549 tumor cells (FR negative). Later a pH‐responsive small‐molecule prodrug **11** containing folate and DOX could self‐assemble into micellar nanoparticles.^[^ ^59^ ^]^ Treatment with such pH‐responsive prodrug nanoparticles substantially improved anticancer efficacy. In addition to targeted drug release, the induced disassembly of prodrug nanoparticles can “switch on” the fluorescence of DOX in the tumor microenvironment for tumor imaging. These studies have established the viability of remarkable nanoplatforms for effective cancer theranostics. Based on the same targeting molecule, an acid‐responsive amphiphilic DOX polymeric prodrug **12** was designed.^[^ ^60^ ^]^ Such FA‐conjugated prodrugs exhibit superior selectivity and cytotoxicity to KB tumor cells over A549 tumor cells. Moreover, this micelle‐based prodrug nanosystem has an optimal diameter of ≈40 nm with desirable stability in an aqueous solution, which is beneficial for long blood circulation and efficient extravasation from tumor blood vessels.

### 4.2 Integrin Targeting

Targeting ligands not only substantially improves drug accumulation but also reduce off‐target side effects. For example, despite the fact that metallodrug‐based theranostic agents have favorable redox and photophysical properties for tumor therapies and bioimaging, the potential toxicity and undesirable biodistribution profile of such theranostic agents still pose great challenges for their in vivo application.^[^ ^61^ ^]^ Therefore, conjugating metallodrugs with targeting moieties is particularly promising for improving tumor therapy. For example, a tumor‐targeting bioresponsive prodrug **13** that contains an RGD peptide and a benzimidazole‐coordinating ruthenium (Ru) was designed (Figure 4b).^[^ ^56^ ^]^ This prodrug can efficiently accumulate in cervical tumor tissues and subsequently release the therapeutic drugs activated in the acid environment. Additionally, this complex was able to emit a deep‐red luminescence, which allows the prodrug system to be used in tumor imaging and specific staining of clinical specimens, highlighting the potential of these metallodrug‐based theranostic agents.

## 5 Fluorescent Prodrug Systems for Breast Cancer

Breast cancer, the most common type of female cancer, has become the second leading cause of cancer‐related deaths among women worldwide.^[^ ^62^ ^]^ Because breast cancer is associated with the steroid hormone estrogen, the discovery of the estrogen receptor (ER) provided not only a powerful predictive and prognostic marker but also an efficient target for the treatment of breast cancer.^[^ ^63^ ^]^ In addition, the CD44 receptor,^[^ ^64^ ^]^ TfR,^[^ ^65^ ^]^ carbonic anhydrase IX (CAIX),^[^ ^66^ ^]^ mannose receptor,^[^ ^67^ ^]^ FA receptor,^[^ ^68^ ^]^ and D‐glucose transported protein^[^ ^69^ ^]^ overexpressed in breast tumor tissues can serve as effective targets for improving tumor therapy (**Table** **4**).

**Table 4:** Targeted fluorescent prodrugs for breast cancer

Target	Fluorophore	Visible mechanism	Specificity	Refs.
ER	DOX	Fluorescence labeling	MCF‐7 tumor	[82]
	Fluorescent ligand	TR‐FRET	MCF‐7 tumor	[83]
	phthalocyanine	Fluorescence & Photodynamics	MCF‐7 tumor	[84]
	pyropheophorbide‐a	Fluorescence & Photoacoustic	4T1 breast tumor	[85]
	Phospholipid	Enzyme triggering	MCF‐7 breast cancer cells	[86]
	Cy5.5	FRET	MCF‐7 breast cancer cells	[87]
	coumarin	ICT	MCF‐7 breast cancer cells BT‐474	[88]
	SN38 conjugate	ICT	4t1 breast cancer cells	[89]
	TPE	AIE	MCF‐7 breast cancer cells	[90]
	resorufin	ICT	MCF‐7 breast cancer cells	[91]
	ICG	Fluorescence labeling	MCF‐7 breast tumor	[92]
	Alexa Fluor 647 succinimidyl ester (AX)	Fluorescence labeling	MCF‐7 breast tumor	[93]
	protoporphyrin IX (PpIX)	Fluorescence labeling	MCF‐7 breast cancer cells	[94]
	Cy5.5	FRET	MCF‐7 breast cancer cells	[95]
CD44	Cy7	Fluorescence labeling	MDA‐MB‐231 cells	[78]
	Cy3	Fluorescence labeling	MDA‐MB‐231 tumor	[96]
	FITC	Fluorescence labeling	MCF‐7 cells	[97]
	FITC and rhodamine	FRET	4T1 tumor	[98]
	Cy5.5	Fluorescence labeling	4T1 cells	[99]
	Curcumin	Fluorescence labeling	MDA‐MB‐231 cells	[100]
	Cy5.5	Fluorescence labeling	MDA‐MB‐231 tumor	[101]
Transferrin	DiR	Fluorescence labeling	MCF‐7/ADR tumor	[102]
	Fluorescein isothiocyanate	Fluorescence labeling	MCF‐7 tumor	[103]
	Dox	Fluorescence tracking	MCF‐7 cells	[104]
CAIX	BODIPY	Fluorescence labeling	MCF‐7 tumor	[79]
	Naphthalimide	Fluorescence labeling	MDA‐MB‐231 cancer stem cells tumor	[81]

### 5.1 Estrogen Receptor Targeting

**Figure 5:**
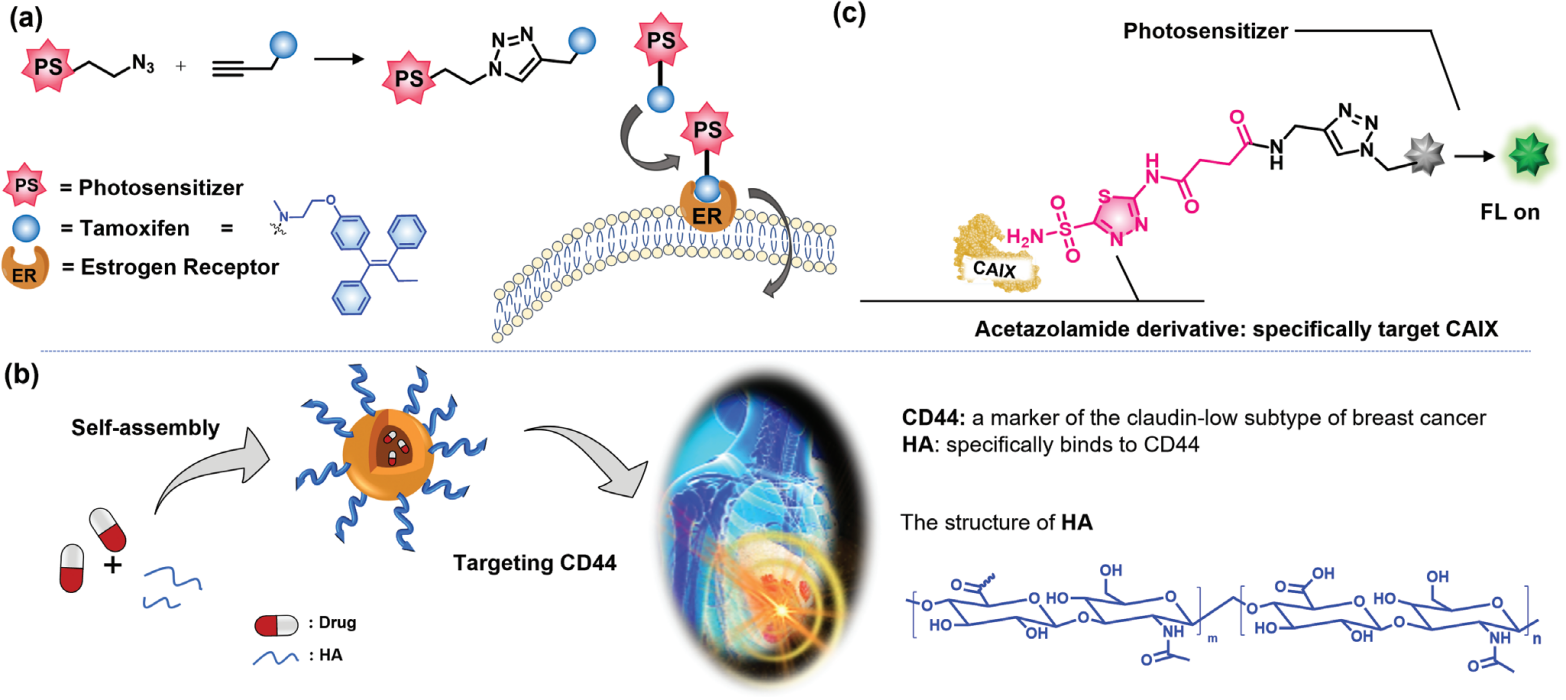
Design of ER a), CD44 b), and CAIX‐targeted c) fluorescent prodrugs for breast cancer therapy.

Tamoxifen, a pioneering drug as a selective estrogen receptor modulator, has been used as the standard of care for advanced ER‐positive breast cancer and developed as a selective targeting agent for breast tumor treatment (**Figure** **5**a).^[^ ^70^ ^]^ For example, an ER‐targeted photosensitizer containing a tamoxifen‐modified Ru (II) polypyridyl complex that demonstrated enhanced cellular uptake and PDT efficacy against ER‐overexpressing breast cancer cells was reported.^[^ ^71^ ^]^ Moreover, the prodrug system can be activated by two‐photon excitation to generate cytotoxic ^1^O_2,_ damaging intracellular lysosomes and leading to cell death. In a later study, the same group developed a NIR activatable small‐molecule photosensitizer that could effectively target the ERs of breast cancer cells,^[^ ^72^ ^]^ thereby providing high accuracy and efficiency for treating ER‐overexpressing breast cancer cells. In the same year, a unimolecular O_2_^•−^ generator that targets mitochondrial respiration (based on the antiestrogenic drug tamoxifen, capable of enhanced hypoxic tumor phototherapy) was designed and synthesized.^[^ ^73^ ^]^ This prodrug not only exhibited superior antihypoxia ability but also realized the accurate tumor diagnosis and the identification of tumor tissue boundary in a mouse model.

### 5.2 CD44 Receptor Targeting

CD44, a glycoprotein on the surface of the cell, participates in immune recognition, cell‐to‐cell and cell‐to‐matrix interactions, cell migration, and other processes.^[^ ^74^ ^]^ HA is a promising ligand for targeting CD44‐overexpressing breast cancer cells, such as MDA‐MB‐231 cells (Figure 5b). However, HA‐modified drug carriers accumulate preferentially in the liver after systemic injection, thereby resulting in insufficient accumulation in tumor tissues. The modification of phosphorylcholine (PC) improves the stability and biocompatibility of HA‐conjugated nanoparticles.^[^ ^75^ ^]^ Based on this finding, PC‐modified HA prodrug micelles, which can respond to both acidic and reducing environmental conditions and selectively inhibit cancer cells were reported.^[^ ^76^ ^]^ The release of DOX from such prodrug micelles was much higher at endosomal pH and in a reducing environment (10 mm dithiothreitol) than in a normal physiological environment. Moreover, the results indicated that the uptake of DOX from HA prodrug micelles was higher in MDA‐MB‐231 cells with overexpressed CD44 receptors. Subsequently, the same group synthesized a pH‐responsive prodrug based on the aggregation‐induced emission (AIE) effect, which could self‐assemble into micelles in an aqueous solution.^[^ ^77^ ^]^ After treatment with the micelles, the CD44‐overexpressing MDA‐MB‐231 cancer cells displayed a much stronger fluorescence than NIH3T3 cells did, suggesting that the modification of HA significantly improved the accumulation of prodrug.

### 5.3 Transferrin Receptor Targeting

Similar to liver cancer, TfR is overexpressed in breast tumor tissues. Therefore, modification of the Tf targeting ligand can improve the accumulation of therapeutics and the efficacy of breast cancer treatment. For instance, the Tf‐based nanocarriers containing PTX prodrug, which was labeled with FITC or ICG‐Der‐02, a NIR dye, for imaging and targeted delivery of PTX was reported.^[^ ^78^ ^]^ In vitro evidence indicated that the prodrug efficiently inhibited tumor cell proliferation and overcame PTX resistance in MDA‐MB‐231 cells, superior to the effects of free PTX treatment.

### 5.4 Carbonic Anhydrase IX Targeting

Acetazolamide (AZ), a well‐known inhibitor of carbonic anhydrase IX (CAIX), can specifically target CAIX. For instance, an AZ‐modified BODIPY photosensitizer could combine the advantages of the antiangiogenesis therapy and PDT, which mitigated the effects of PDT‐based hypoxia (Figure 5c).^[^ ^79^ ^]^ This prodrug showed a specific affinity to aggressive cancer cells (MDA‐MB‐231 cells) overexpressing CAIX derived from breast cancer patients.^[^ ^80^ ^]^ This study provides an attractive therapeutic approach for targeting CAIX‐overexpressing tumors. Recently, this group reported a small‐molecule construct that promised effective therapeutic targeting of breast cancer stem cells (CSCs) in the refractory hypoxic tumor environment.^[^ ^81^ ^]^ The prodrug containing AZ promoted cancer‐specific localization in MDA‐MB‐231 CSCs. Furthermore, CAIX‐assisted selective uptake of the compound was validated by CAIX gene silencing. The experimental results demonstrated it could serve as a hypoxia‐labilemolecular platform to selectively target breast CSCs, decrease CSC migration and tumorigenesis rates and reduce tumor growth. This preclinical study presents the first CSC‐targeting small molecule to prevent tumorigenesis in an animal model.

## 6 Fluorescent Prodrug Systems for Glioblastoma

Glioma, initiated in the glial cells of brain or spine, accounts for ≈80% of all malignant brain tumors.^[^ ^105^ ^]^ The most significant challenges to the clinical treatment of glioma are the existence of physiological barriers such as the blood–brain barrier (BBB) and the blood‐tumor barrier (BTB) that may impede drug penetration and efficient accumulation in glioma tissues.^[^ ^106^ ^]^ In recent years, the innovative design of fluorescent molecular probes has facilitated their penetration through BBB, providing a new method of treating glioma. In the case of small molecule, liposoluble characteristics and small size (MW < 400 Da) are mostly required to cross BBB by free diffusion.^[^ ^107^ ^]^ Additionally, the surface modification of BBB‐penetrating molecules, peptides, or polymers can enhance the ability of nanoparticles to permeate BBB. Furthermore, developing prodrug systems which can enhance receptor‐mediated endocytosis can penetrate BTB.^[^ ^108^ ^]^ For example, taking advantage of the integrins overexpressed in glioma cells offers the possibility of selective uptake and targeted therapy and improves the accumulation of antitumor drugs in the brain. In addition to integrins, interleukin‐13 receptor *α*2 (IL‐13R*α*2), and neuropilin1 (NRP1), overexpressed in glioma cells (U87MG), have provided attractive targeting sites for improving drug accumulation.^[^ ^109^ ^]^ However, delivery of the prodrug into glioblastoma always has to consider the drug resistance attributed to the activation of drug efflux transporters such as P‐glycoprotein (P‐gp).^[^ ^108^ ^]^The rational design of prodrug systems that covalently conjugate fluorophores and these targeting ligands enables their accumulation and drug release to glioma tumor cells (**Table** **5**).

**Table 5:** Targeted fluorescent prodrugs for glioblastoma

Target	Fluorophore	Visible mechanism	Specificity	Refs.
*α*v*β*3 integrin	Coumarin	Contact‐mediated quenching	Glioblastoma U87 cells	[110]
	5(6)‐carboxylfl uorescein (FAM)	Dual FRET	Glioblastoma U87 cells	[111]
	Tetraphenylsilole (TPS)	Aggregation‐induced emission	Glioblastoma U87 cells	[112]
	IR780	Fluorescence & photodynamics	Glioblastoma U87 cells	[113]
	Naphthalimide	Ratiometric fluorescence	Glioblastoma U87 cells	[114]
Interleukin	Coumarin	Fluorescence labeling	Brain glioma	[115]
	GFP	Fluorescence labeling	Brain glioma	[116]
NRP1	Nile Red	Ratiometric fluorescence	Glioblastoma U87 cells	[117]
	Coumarin	Fluorescence labeling	Brain glioma	[118]
	Cy5.5	Fluorescence labeling	Brain glioma	[119]
	Fluorescein isothiocyanate	Fluorescence labeling	C6 xenograft tumor	[120]
	Fluorescein	Fluorescence labeling	C6 glioma	[121]
	Pyro	Fluorescence & photodynamics	Glioblastoma U87 cells	[122]
CTX	ICG	Ratiometric fluorescence	Brain glioma	[123]
	Cy5.5	Fluorescence labeling	Brain glioma	[124]

### 6.1 Integrin Targeting

**Figure 6:**
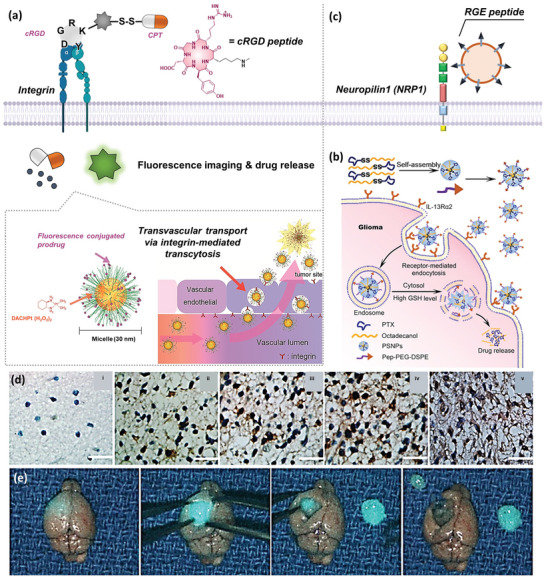
Fluorescent prodrug systems for glioblastoma. a) Schematic illustration of different targeted ligands for glioblastoma of cRGD peptide. Reproduced with permission.^[^ ^128a^ ^]^ Copyright 2013, American Chemical Society. b) Pep‐1 glioma‐homing‐peptide fabricated NPs targeting IL‐13R*α*2. Reproduced with permission.^[^ ^131^ ^]^ Copyright 2017, American Chemical Society. c) Schematic illustration of different targeted ligands for glioblastoma of NRP1. d) Representative sections of IL‐13R*α*2 immunoreactivity in normal brain tissue (i) and different grades of glioma tissues (ii–v). Bar = 25 µm. Reproduced with permission.^[^ ^129b^ ^]^ Copyright 2017, American Chemical Society. e) Real‐time NIR‐imaging by SIRIS and surgical resection of GBM in a mouse model. Reproduced with permission.^[^ ^123^ ^]^ Copyright 2019, Elsevier.

Cell surface integrin can bind to arginine‐glycine‐aspartic acid (RGD) peptide efficiently.^[^ ^125^ ^]^ Glioblastoma (GBM), a subclass of glioma, shows an accumulation of integrins in the transmembrane compared to normal brain cells, and the upregulation of integrin is related to poor prognosis and survival.^[^ ^126^ ^]^ Therefore, in the last decade, attempts have been made to use RGD peptides to improve the glioma targeting of fluorescent molecular probes.^[^ ^110^, ^127^ ^]^ Since the cyclic RGD (cRGD) peptide is most effective at targeting GBM, a cRGD peptide was introduced to a theranostic system for U87 cell targeting.^[^ ^114^ ^]^ Such a prodrug has a GSH‐responsive disulfide bond linking CPT and cyclic RGD peptide. Owing to the changeable electron‐withdrawing effect of two acyls in amine and imine, the redshift of fluorescence (from 473 to 536 nm) can reflect not only the release of CPT but also the receptor‐mediated endocytosis of the drug system. Numerous studies have also shown that this RGD peptide promotes endocytic uptake via integrin receptors.^[^ ^128^ ^]^ For example, a platinum‐based anticancer drug‐incorporating polymeric micelle (PM) with cRGD (cRGD/m) not only exhibited superior GBM targeting ability toward U87MG cells but also penetrated the vascular barrier (**Figure** **6**a).^[^ ^128a^ ^]^ Given that GBM is difficult to approach due to the BBB, relying solely on an EPR effect is not adequate for GBM‐targeted therapy. The in situ translocating behavior of cRGD/m has been demonstrated; cRGD facilitates the internalization of nanomicelles into the cells via integrin‐mediated transcytosis.

### 6.2 Interleukin‐13 Receptor *α*2 (IL‐13R*α*2) Targeting

IL‐13R*α*2, a membrane‐bound protein, is a typical receptor upregulated in glioblastoma but not in normal brain tissues (Figure 6d).^[^ ^129^ ^]^ It shows a high affinity for IL‐13 but has a little function for cellular signaling pathways. Therefore, the potential of IL‐13R*α*2 for use in receptor‐directed cancer therapy has been suggested.^[^ ^130^ ^]^ Pep‐1, a linear homing peptide with an amino acid sequence of CGEMGWVRC, can penetrate the BTB via IL‐13R*α*2‐mediated endocytosis. For example, the theranostic NPs which were synthesized by self‐assembling redox‐responsive PEGylated PTX prodrug (PTX‐SS‐C18) can target U87MG glioma cells (Figure 6b).^[^ ^131^ ^]^ Additionally, the self‐assembled NPs were covered with Pep‐PEG‐DSPE to form Pep‐PSNPs. Another example is coumarin 6‐loaded Pep‐1‐fabricated PEG‐PLGA copolymer NP (Pep‐NP), whose in vivo targeting ability was also demonstrated.^[^ ^115^ ^]^ The nanoparticles exhibited an enhanced and precise targeting efficacy, suggesting their therapeutic potential as a drug delivery platform for the treatment of glioma.

### 6.3 NRP1 Targeting

NRP sare single‐pass transmembrane proteins of GBM tumor cells.^[^ ^132^ ^]^ RGE peptide with the sequence of RGERPPR has been used as an NRP‐targeting ligand and can improve the GBM targeting efficacy of nanoparticles by crossing BBB (Figure 6c). For example, conjugating RGE on the surface of exosomes that were loaded with curcumin (Cur) and superparamagnetic iron oxide nanoparticles (SPIONs) (RGE‐Exo‐SPION/Cur) was advantageous for glioma targeting and therapy.^[^ ^133^ ^]^ Treatment with these exosomes showed remarkable therapeutic efficacy through the synergistic effect of SPIONs‐mediated magnetic fluid hyperthermia and Cur‐mediated chemotherapy. Furthermore, a 2021 study showed that the NIR‐emissive GRE‐modified extracellular vesicles (EVs) can efficiently target glioma.^[^ ^134^ ^]^ In this design, the NIR‐absorbing photothermal agents (ICG) and anticancer drugs (PTX) were coloaded in the EVs (ICG/PTX@RGE‐EV) for combinational cancer therapy and U251 tumor cell imaging.

### 6.4 Glioma‐Specific Chloride ion Channel Targeting

Chlorotoxin (CTX) peptide (which is purified from scorpion toxin) contains 36 amino acids. Such a peptide can target a glioma‐specific GCC expressed on glioma cells, thereby serving as an exceptional targeting ligand to improve the accumulation of drugs or diagnostic agents. For example, attempts have been made to visualize glioma cells and tissue sections on patient biopsies using biotinylated CTX and avidin‐rhodamine conjugate.^[^ ^135^ ^]^ Additionally, a polymeric acid CTX nanoconjugate containing CTX, NIR fluorophore (ICG), and tri‐leucine peptide (LLL) was developed for NIR‐guided GBM resection.^[^ ^123^ ^]^ In this nanoconjugate, poly(*β*‐L‐malic acid) was used as a backbone to connect CTX, ICG, and LLL; LLL was introduced to avoid the self‐quenching of ICG for synchronized NIR imaging‐assisted tumor resection (Figure 6e).

## 7 Fluorescent Prodrug Systems for Colorectal Cancer

CRC is one of the most common types of malignant tumors in humans, with 151 030 estimated new cases of colorectal cancer and 52 580 deaths in the United States alone in 2022, and its incidence is increasing especially among young adults.^[^ ^136^ ^]^ Since the intestinal microflora is in direct contact with the colonic cells, the enzymes of the bacterial microflora may play a pivotal role in colon carcinogenesis.^[^ ^137^ ^]^ Therefore, enzymes produced by colonic anaerobic microflora, such as azoreductases, could serve as targets to improve the selectivity for colorectal cancer. In addition, hyaluronic acid (HA) receptors, such as CD44, are overexpressed on the surface of colonic cancer cells and could therefore be used as targets for colorectal cancer treatment (**Table** **6**).^[^ ^138^ ^]^

**Table 6:** Targeted fluorescent prodrugs for colorectal cancer

Targets	Targeted units	Targeted cells	Fluorophores	Activator	Refs.
Azoreductase	Azo	Hep G2 cell	Cy (NIRF)	Azoreductase	[141]
HA receptors	HA	CT26 cells	Pheophorbide A	NIR laser	[142]
		HCT‐116	Naphthalimide	Thiols	[143]

### 7.1 Azoreductase Targeting

**Figure 7:**
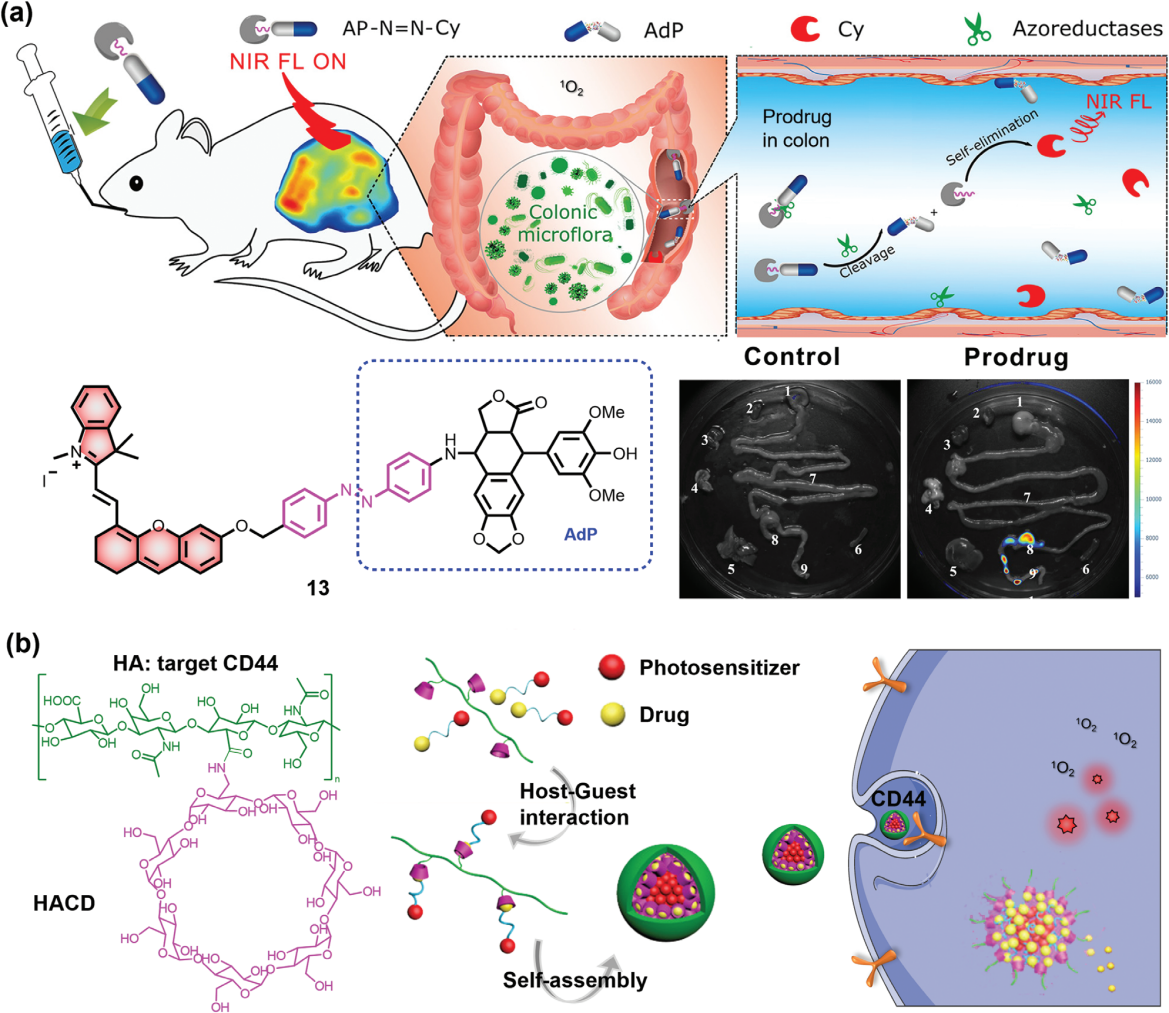
Fluorescent prodrug systems for colorectal cancer. a) Schematic illustration and chemical structure of fluorescent prodrugs that target azoreductases. Reproduced with permission.^[^ ^141^ ^]^ Copyright 2020, American Chemical Society. b) Schematic illustration of fluorescent prodrugs that target CD44 receptor for colorectal cancer. Reproduced with permission.^[^ ^142^ ^]^ Copyright 2020, Wiley.

The azobenzene moiety, which can be activated by azoreductases, is one of the most common ligands to be incorporated into colon‐specific prodrugs.^[^ ^139^ ^]^ Therefore, using an azoreductase‐responsive prodrug is a promising approach for the targeted treatment of colon‐related diseases. Although numerous azoreductase‐responsive prodrugs (olsalazine, sulfasalazine, and balsalazide) have reached the clinic,^[^ ^140^ ^]^ the development of fluorescent prodrugs that can be activated by azoreductases remains difficult. A novel colon‐specific prodrug 13 that can real‐time monitor the release of AdP has been commonly used as a drug for the therapy of colon cancer (**Figure** **7**a).^[^ ^141^ ^]^ In this design, the fluorescence signal of the prodrug would change by introducing an azobenzene group; the azobenzene group can serve as a switch to “turn on” the fluorescence of Cy, which is caused by the self‐elimination reaction of the cleavage of an azo bond. Additionally, such a prodrug system exhibited both high stability and low toxicity before reaching the colon tissues. Importantly, researchers can use fluorescence imaging to precisely track drug delivery both in vitro and in vivo, since drug release led to fluorescence. Of note, this study is the first demonstration that colon‐specific drug release and biodistribution can be tracked via in vivo real‐time multimodal imaging.

### 7.2 HA Receptor Targeting

A 2020 study showed that a supramolecular nanocomplex containing HA, Pheophorbide A (PPa) and NLG919 could serve as a targeted fluorescent prodrug for combination immunotherapy (Figure 7b).^[^ ^142^ ^]^ In this system, PPa and NLG919 served as a photosensitizer and an inhibitor of indoleamine 2,3‐dioxygenase 1 (IDO‐1), respectively. Importantly, the combination of photodynamic therapy and IDO‐1 inhibitor can effectively eliminate CT26 colorectal tumors in immunocompetent mice. In addition, HA could serve as a targeting ligand by recognizing the CD44 receptor that is highly expressed on the surface of cancer cells. Based on the same targeting strategy, an enzyme‐activated supramolecular assembly system that connects adamantane‐naphthalimide, fluorescent CPT prodrug, and *β*‐CD‐modified HA by disulfide bonds was synthesized.^[^ ^143^ ^]^ The supramolecular polysaccharide assembly possessed low cytotoxicity, and showed targeted cellular imaging and controllable drug release with spatial accuracy, thus establishing an innovative platform for monitoring the drug delivery in a real‐time manner.

## 8 Conclusion and Perspectives

Fluorescent targeting prodrugs features the visible cancer cell‐specificity and amplified the medicine differential between cancer and normal cells. So far, the fluorescent targeting prodrugs have been widely studied in lung cancer (biotin/CD56/EGFR), liver cancer (ASGPR/GAR/SP94/TfR), cervical cancer (FR/integrin), breast cancer (ER/CD44/TfR/CAIX), glioma (integrin/IL‐13R*α*2/NRP1/CTX), and colorectal cancer (azoreductase/HA receptors). Receptors are mainly responsible for targeting specific cells, and in some cases, they also take part in the fluorescent mechanism. Fluorophores are mainly involved with coumarin, ethidium, BODIPY, Tetraphenylene, ICG, Quercetin, CPT, Cy‐n, metal‐complex, TPE, naphthalimide, rhodamine, GFP, fluorescein, etc. Two types of logical relations between fluorophore and drug and even receptor were included: i) molecular intervention; ii) labeling. The former results in changeable fluorescence via mechanisms like Fluorescence Resonance Energy Transfer (FRET), Intromolecular Charge Transfer (ICT), Photoinduced Electron Transfer, and AIE, through the linker or the definite space; the latter can provide continuous fluorescence tracking. Fluorescent prodrugs could be activated by specific stimuli, such as endogenous biomolecule‐promoted reactions, enzymes, or light. The trigger in prodrugs always plays a dual function including adjustment of fluorescence and inactivation of a drug.

Despite the breakthroughs represented by the development of targeted fluorescent prodrugs, their use in clinical settings still faces many challenges. From our viewpoint, the following aspects should be further considered sequentially to support the future development of targeted fluorescent prodrugs:

1) Targeting efficiency. The efficiency of tumor cell targeting of fluorescent prodrugs remains unsatisfactory, especially for small‐molecule drugs in the human body. To address this challenge, the large‐scale screening of cell‐specific receptors, targets, or inhibitors that can be efficiently targeted by fluorescent prodrugs is of particular promise for precision medicine. Indeed, the uniqueness of targeted fluorescent prodrugs is that one can track drug distribution in real‐time. Thus, the fluorescent properties of rationally designed prodrugs may serve as convenient yet high‐throughput visible platform in screening experiments.
2) Microenvironment recognition. Even if targeting efficiency is improved, the heterogeneous microenvironment of tumor tissues remains an obstacle to precise treatment. Indeed, the microenvironment of tumor tissue in various organs is different, and this is further complicated when considering the tumor at different stages. For example, the differences between cancer stem cells (CSCs) and regular cancer cells make CSC targeting even more important, especially when the goal is the prevention of relapse. Consequently, constructing prodrugs that can be triggered specifically by the cancerous microenvironment, preferably with a visible fluorescent response toward this microenvironment, is promising for precision medicine.
3) Drug resistance. Even if precise delivery and release of targeted fluorescent prodrugs are realized, the anticancer efficacy of drug platforms still suffers from drug resistance. Of particular promise, many therapeutic strategies, such as chemical interference in CSCs, apoptosis dysregulation, autophagy, efflux transporters, or gene mutation can reverse the drug‐resistant microenvironment. Given this paradigm, integration of these therapeutic strategies to the targeted fluorescent prodrugs will produce multifunctional prodrug systems that can monitor the modulation of the tumor microenvironment and drug release. Consequently, we believe that targeted fluorescent prodrugs can provide a wealth of convincing first‐hand information for precision medicine. Although it is challenging to rationally design an individual prodrug possessing these essential properties simultaneously, we envision that our improved understanding of cancer cell‐specific surface receptors gained over recent decades, in combination with the established success of various targeting strategies, will accelerate the clinical translation of fluorescent prodrug systems for organ‐specific cancer therapies. We expect fluorescent prodrug systems to serve as the next generation of “VIPs”—Very Important Prodrugs.

## Conflict of Interest

The authors declare no conflict of interest.
